# Impact of recreational football in men with prostate cancer undergoing androgen deprivation therapy

**DOI:** 10.1002/bco2.70008

**Published:** 2025-04-10

**Authors:** Sofia Mesquita, Susana Póvoas, Diogo Nunes‐Carneiro, David Sá‐Couto, Mário Santos, Avelino Fraga, Miguel Silva‐Ramos

**Affiliations:** ^1^ Department of Urology Unidade Local de Saúde de Santo António Porto Portugal; ^2^ Research Center in Sports Sciences, Health Sciences and Human Development (CIDESD) University of Maia Maia Portugal; ^3^ Department of Sports Science and Clinical Biomechanics, SDU Sport and Health Sciences Cluster (SHSC) University of Southern Denmark Odense Denmark; ^4^ Department of Cardiology Unidade Local de Saúde de Santo António Porto Portugal; ^5^ Instituto de Ciências Abel Salazar (ICBAS) University of Porto Porto Portugal

**Keywords:** androgen deprivation therapy, cardiovascular, physical functioning, prostate cancer, recreational football

## Abstract

**Objectives:**

To evaluate the effects of supervised recreational football‐based exercise intervention on physical functioning, cardiovascular and metabolic health, bone strength and quality of life in men with PCa receiving ADT.

**Materials and Methods:**

Men with locally advanced or metastatic PCa undergoing ADT were allocated to a supervised recreational football‐based exercise programme. Patients were invited to participate in 2–3 one‐hour weekly sessions for 8 months and encouraged to participate in at least 2 session/week. Outcomes were physical functioning (postural balance, agility, muscle strength and aerobic capacity), blood pressure, lipid profile (LDL‐cholesterol, HDL‐cholesterol, total‐cholesterol and triglycerides), glucose, glycated haemoglobin (HbA1C) and high sensitivity C‐reactive protein (CRP), proximal femoral and lumbar spine (L2‐L4) bone mineral density (BMD) and quality of life (EORTC QLQ‐C30 and QLQ‐PR25). Descriptive and inferential statistical analysis was performed using the IBM® SPSS® Statistics version 29 software.

**Results:**

From September to October 2022, 23 interested patients were screened and 12 were included and completed the supervised recreational football‐based exercise programme. A significant improvement in aerobic capacity was recorded, with the patients improving the distances walked over 6 minutes (580.0 vs. 537.5, *p* = 0.005). There was a significant reduction in systolic blood pressure from a median of 134 to 123 and a non‐statistically significant decrease in diastolic blood pressure from a mean of 80 to 73 mmHg. There were no significant differences for lumbar and femoral BMD. A tendency for higher general health status post‐intervention (80.6 vs. 70.8, p = 0.094) and a significant difference in the cognitive domain (83.0 vs. 100.0, p = 0.020) were recorded.

**Conclusions:**

Men with PCa undergoing ADT experience side effects that, when combined with the physical inactivity and poor fitness often seen in these patients, may heighten their risk of cardiovascular and metabolic complications. A recreational football‐based exercise programme can be implemented to improve physical fitness, aerobic capacity, systolic blood pressure and quality of life.

## 1 INTRODUCTION

Prostate cancer (PCa) is the second most diagnosed cancer in men.^1^ Androgen deprivation therapy (ADT) remains a fundamental component in the management of PCa, as up to half of men diagnosed with PCa will undergo ADT at some point of their treatment course.^2^ Men with PCa commonly face adverse effects linked to both the disease and its treatment, however, including urinary incontinence, erectile dysfunction,^3^ fatigue, anxiety and depression,^4^ and diminished health‐related quality of life.^5^ Moreover, the severe hypogonadal condition induced by ADT is associated with loss of lean body mass, increased fat mass, insulin resistance, decreased bone mineral density, increased risk of fractures and poor functional performance.^6^ The combination of ADT‐induced adverse effects and subsequent changes in health behaviour, such as physical inactivity and poor physical fitness could potentially predispose PCa patients to significant morbidity, including increased risk of cardiovascular and metabolic complications, leading to increased mortality.^7^

Therefore, strategies targeting the mitigation of ADT‐induced adverse effects could yield significant survival benefits for patients with PCa.

Supervised aerobic and resistance exercise training of a moderate intensity has proven effective in improving muscle mass, strength, physical function and balance in hypogonadal men.^8^, ^9^ Such interventions have also reported clinically relevant improvements in disease‐specific quality of life in men on long‐term ADT.^5^ This exercise frequently faces challenges in engaging most of the population and sustaining long‐term adherence to exercise. Recreational football is an alternative to this conventional exercise programmes. The popularity of recreational football, coupled with its physical and physiological demands, including high cardiovascular, metabolic and musculoskeletal stress, has supported the implementation of exercise programmes based on this modality for men with PCa undergoing ADT^10^, ^11^, ^12^. These programmes have shown to be effective in improving lean body mass and muscle strength as well as bone mineral density (BMD) in this population^11^, ^12^

In men undergoing ADT, sexual function is affected by various treatment‐related factors, primarily manifested by reduced libido, erectile dysfunction and testicular atrophy. Furthermore, physical effects, such as undesirable changes in body composition, psychological distress and decreased functional capacity also contribute to sexual dysfunction.^13^ Additionally, exercise in a group‐based setting contributes to the acceptance of sexual changes by reinforcing strength‐based aspects of masculinity and peer support.^14^

The PCaGoal programme was designed to assess the potential effect of supervised recreational football training on physical functioning, cardiovascular and metabolic profile, bone health and quality of life in men with PCa managed with ADT.

## 2 MATERIALS AND METHODS

### 2.1 Study design and population

The PCaGoal programme is a single‐arm trial aimed at men with locally advanced or metastatic PCa undergoing ADT allocated to a supervised recreational football‐based exercise intervention. The study was conducted from September 2022 to August 2023. The study was approved by the Ethics Committee of Education, Training and Research department at Unidade Local de Saúde de Santo António, Porto, Portugal.

Patients were recruited based on outpatient evaluation records and data from the multidisciplinary consultation.

Eligible participants were men diagnosed with prostate adenocarcinoma undergoing ADT for a minimum of 6 months and Eastern Cooperative Oncology Group Performance Status (ECOG PS) grade of 0 or 1. The criteria for exclusion were age above 79, critical coronary artery disease, myocardial infarction or unstable angina in the last 6 months, severe chronic obstructive pulmonary disease (GOLD stage III/IV), severe congestive heart failure (NYHA class III/IV) and physical limitations that impair running and/or kicking a football ball.

After initial recruitment, all patients were submitted to an initial medical assessment phase. A cardiovascular evaluation was carried out, with clinical evaluation, a transthoracic echocardiography and an exercise stress test.

### 2.2 Intervention

Patients were invited to participate in 2–3 one‐hour weekly sessions for 8 months and encouraged to participate in at least 2session/week. The training sessions took place at a local football club. Each 60‐min session comprised a 15‐min warm‐up, followed by 11 min of football drills and 3 × 10‐min of recreational football training played as small‐sided games with 2‐min breaks, in a 40 × 20‐m artificial grass pitch. In the first half (4 months) of the intervention, the participants performed walking football, with running being allowed after that time point on. Sessions were delivered by a Physical Education and Sport Sciences graduated and supervised by the research team.

The training sessions were monitored for internal (ratings of perceived exertion and cardiovascular load, using heart rate monitors – Firstbeat Technologies Ltd., version 4.7.2.1, Jyväskylä, Finland) and external load markers (locomotor activity pattern, using global position system – Catapult MinimaxX S4, Catapult Sports, Canberra, Australia) to characterise the physical and physiological demands of the activity. To help improve motivation and adherence, a behavioural component during the training session included exploring with patients' barriers to exercise and strategies to help them incorporate regular physical activity in their lives was provided.

### 2.3 Outcomes

Patients underwent two main outcome assessments – at baseline (before the onset of the programme) and a final assessment after 8 months. Additionally, physical performance was reassessed after 4 months into the programme. Our reporting exclusively includes outcomes from patients who completed all assessments.

The primary outcome of the study was physical functioning. Postural balance was assessed using a single‐leg balance test. Agility was evaluated using the sit‐to‐stand test. Functional capacity was estimated using the 6‐minute walk test (6MWT).^15^ Detailed descriptions of the tests can be found in the eMethods section.

The European Organisation for Research and Treatment of Cancer (EORTC) QLQ‐C30^16^ and QLQ‐PR25^17^ was used to assess cancer‐specific quality of life status. Comprehensive details of the questionnaires are also available in the eMethods section.

Blood pressure was measured with a digital sphygmomanometer. Both measures were taken before the initial and final physical functioning assessments after each patient had seated for 10 minutes.

Blood samples for the measurement of lipid profile (LDL‐cholesterol, HDL‐cholesterol, total cholesterol and triglycerides), glucose, glycated haemoglobin (HbA1C) and high‐sensitivity C‐reactive protein (CRP) were collected from each patient in the morning after at least 12 h of fasting. Assessment of total body lean mass (g), fat mass (g), fat mass percentage and proximal femur and lumbar spine bone mineral density (BMD) (g/cm2) were derived from dual‐energy X‐ray absorptiometry (DXA) scan (Horizon DXA System).

Attendance and reasons for nonattendance of training sessions were recorded in a training logbook, as well as serious adverse events occurring during training were reported. We defined a reasonable level of feasibility as attending 50% or more of the training sessions throughout the programme.

### 2.4 Data analysis

Statistical analysis was performed using the IBM® SPSS® Statistics version 29 software. Results for continuous variables were expressed as mean ± standard deviation or as median (interquartile range) according to its distribution. Categorical variables were described as absolute and relative frequencies. The comparison of baseline and post‐intervention results was performed by paired two‐sample t‐test and Wilcoxon signed‐rank test. The level of statistical significance adopted was p < 0.05.

## 3 RESULTS

### 3.1 Participants

From September to October 2022, 23 interested patients were screened for eligibility and 12 were included and completed the supervised recreational football‐based exercise programme. The remaining patients declined participation in the programme for the following reasons: geographic distance/travel time (n = 5), incompatible schedule (n = 3), poor physical condition (n = 2) and a fracture secondary to a fall during recruitment (n = 1).

Patient characteristics are presented in Table 1. The mean age of patients was 72.1 (3.4). At baseline, the median time on ADT was 23.0 months (7.0–47.5). Bone metastases were presented in five patients. None of these patients exhibited symptomatic disease that would render them ineligible for training.

**TABLE 1:** Baseline characteristics of patients.

Age, years – mean (SD)	72.1 (3.4).
BMI, kg/m^2^ – mean (SD)	26.6 (2.2)
Time since diagnosis, years –	6.2 (4.2)
ISUP Gleason Grading at diagnosis – n (%)	
Group 2	3 (25.0)
Group 3	5 (41.7)
Group 4	3 (25.0)
Group 5	1 (8.3)
Previous treatment at baseline – n (%)	
Radical prostatectomy	7 (58.3)
ADT and radiation therapy	2 (16.7)
Radiation therapy	7 (58.3)
Chemotherapy	3 (35)
Current treatment at baseline – n (%)	
aLHRH	9 (75.0)
aLHRH and new androgen receptor pathway inhibitors	3 (25.0)
Time on ADT – median (IQR)	23.0 (7.0–47.5)
Indications for ADT– n (%)	
PSA‐only recurrence after treatment with curative intent	4 (33.3)
Non‐metastatic castrate‐resistant disease	1 (8.3)
Hormone‐sensitive metastatic disease	7 (58.3)
Comorbidities – n (%)	
Cardiovascular disease	6 (50.0)
Hypertension	8 (66.7)
Dyslipidemia	8 (66.7)
Diabetes	5 (41.7)
Depression	3 (25.0)

ADT, androgen deprivation therapy; aLHRH, luteinising hormone‐releasing hormone agonists; BMI, body mass index; IQR, interquartile range; SD, standard deviation.

### 3.2 Adherence and safety

During the programme, 80 football sessions were conducted. Patients attended 83% (71–90) of the training sessions or 2.0 (0.5) training sessions/week.

The median number of falls was 5.0 (3.0–5.5) per person.

There were 10 injuries reported during 738 hours of training (13.6 lesions/1000 h). One rib fracture was reported. The patient resumed football training after conservative treatment.

### 3.3 Physical functioning

Significant differences were observed in postural balance between the baseline assessment and those conducted at 4 and 8 months (Table 2). The mean number of falls before completing 1 minute in balance decreased from 19.8 (8.8) to 11.6 (4.9) at 8 months (p < 0.001). Agility also showed significant differences between the baseline assessment, the 4‐month assessment and post‐intervention (Table 2). The sit‐to‐stand performance improved from baseline to the 4‐month assessment (16.7 vs. 20.6 repetitions, *p* = 0.003) and post‐intervention (16.7 vs. 23.7 repetitions, *p <* 0.001).

**TABLE 2:** Physical functioning.

Variable	N	Baseline	4 months	8 months
**Postural balance**				
Single leg postural balance, sec – median (IQR)	12	17.6 (10.7–41.3)	30.0 (13.0–56.0) * *p* = 0.028	43.5 (33.7–60.0) * *p* = 0.007 ° *p* = 0.008
30″ flamingo test, n – mean (SD)	12	11.3 (4.0)	7.0 (2.8) * *p* = 0.001	5.1 (2.6) * *p <* 0.001 ° *p* = 0.047
60″ flamingo test, n – mean (SD)	12	19.8 (8.8)	13.7 (4.1) * *p* = 0.011	11.6 (4.9) * *p <* 0.001 ° *p* = 0.116
**Agility,** sec – mean (SD)	12	5.8 (1.1)	4.9 (0.6) * *p* = 0.002	4.4 (0.5) * *p <* 0.001 ° *p <* 0.001
**Muscle strength**				
Sit‐to‐stand test, n – mean (SD)	12	16.7 (2.8)	20.6 (4.7) * *p* = 0.003	23.7 (5.8) * *p <* 0.001 ° *p =* 0.051
**6‐minute walk test**				
Distance, m – median (IQR)	10	537.5 (498.8–555.0)		580.0 (572.5–641–3) * *p* = 0.005
Global RPE (0–10, AU) – median (IQR)	10	2.0 (1.8–5.5)		4.0 (3.8–5.5) * *p* = 0.071
Respiratory RPE (0–10, AU) – median (IQR)	10	2.5 (1.8–5.0)		4.0 (1.8–4.0) * *p* = 0.490
Muscular RPE (0–10, AU) – median (IQR)	10	2.5 (1.0–5.8)		4.0 (4.0–6.0) * *p* = 0.119
Maximal heart rate, bpm	10	115 (102–142)		141 (132–148) * *p* = 0.012
Recovery heart rate at 30″, bpm	10	102 (86–125)		117 (104–134) * *p* = 0.025
Recovery heart rate at 60″, bpm	10	102 (83–107)		106 (93–123) * *p* = 0.074
Recovery heart rate at 120″, bpm	10	96 (81–101)		106 (93–123) * *p* = 0.866
**Daily activity**				
Physical activity per week, MET‐min – median (IQR)	12	2520 (1020–7640)	2940 (675–6620) * *p* = 0.213	1360 (0–6720) * *p* = 0.197 ° *p* = 1.00
Sedentary time per day, min – median (IQR)	12	210.0 (157.5–540.0)	180.0 (112.5–180.0) * *p* = 0.102	330.0 (195.0–502.5) * *p* = 0.313 ° *p* = 0.011

AU, Arbitrary Units; bpm, beats per minute; IQR, interquartile range; RPE, Rate of perceived exertion; MET, metabolic equivalent of task; SD, standard deviation.

* from baseline.

° from 4 months.

The 6MWT distance was significantly higher at 8 months compared to the baseline assessment (580.0 vs. 537.5, *p* = 0.005). Patients reached a significantly higher maximum heart rate (141 vs. 115, p = 0.012) with no significant difference observed in the rating of perceived exertion measured.

Regarding self‐reported physical activity behaviour, there were no differences in metabolic equivalent of task (MET) values between assessments.

### 3.4 Blood pressure

There was a significant decrease in systolic blood pressure (133.5 vs. 123.0, p = 0.037) and mean blood pressure (97.0 vs. 93.5, p = 0.020). Although diastolic blood pressure tended to be lower post‐intervention (79.5 vs. 73.2), this difference did not reach statistical significance (p = 0.078).

### 3.5 Blood parameters

There were no differences in lipid profile, glucose control and high‐sensitive CRP (Table 3).

**TABLE 3:** Cardiovascular, metabolic and bone health markers.

Variable	N	Baseline	8 months	*p*
Systolic blood pressure, mmHg – median (IQR)	12	133.5 (124.5–144.8)	123.0 (117.8–133.5)	0.037
Diastolic blood pressure, mmHg – mean (SD)	12	79.5 (10.6)	73.2 (6.4)	0.078
Mean blood pressure, mmHg – median (IQR)	12	97.0 (93.0–100.0)	93.5 (84.0–95.5)	0.020
Resting heart rate, bpm – mean (SD)	12	69.0 (8.9)	67.6 (10.5)	0.611
Total cholesterol, mg/dL – mean (SD)	12	177.9 (40.2)	178.9 (41.2)	0.804
Cholesterol‐LDL, mg/dL – mean (SD)	12	100.6 (35.8)	100.8 (36.3)	0.928
Cholesterol‐HDL, mg/dL – mean (SD)	12	50.0 (8.3)	51.3 (12.2)	0.530
Triglycerides, mg/dL – mean (SD)	12	137.2 (71.7)	130.8 (55.4)	0.607
High‐sensitive CRP, mg/L – median (IQR)	12	2.3 (0.5–3.7)	1.2 (0.5–2.4)	0.929
Glucose, mg/dL – median (IQR)	12	97.0 (87.5–108)	96.5 (88.3–140.5)	0.444
Haemoglobin A1C, % – median (IQR)	12	5.6 (5.2–6.8)	5.8 (5.4–7.3)	0.051
Lumbar BMD (g/cm^2^) – mean (SD)	12	0.95 (0.13)	0.96 (0.13)	0.626
Femoral BMD (g/cm^2^) – mean (SD)	6	0.95 (0.07)	0.95 (0.08)	0.596
Lean body mass (kg) – mean (SD)	6	48.78 (46.20)	48.66 (40.30)	0.929
Fat mass (kg) – median (IQR)	6	21.90 (17–52–31.95)	20.76 (20–76–31.25)	0.600
Fat mass (%)– mean (SD)	6	31.42 (6.69)	31.43 (4.45)	0.989

BMD – bone mineral density; CRP, C‐reactive protein; HDL, high‐density lipoprotein; IQR, interquartile range; LDL, low‐density lipoprotein; SD, standard deviation.

### 3.6 Bone health and body composition

There were no significant differences for lumbar and femoral BMD (Table 3).

No significant differences in lean body mass from baseline to 8 months were found (p = 0.929). Similarly, no significant differences were observed in fat mass (p = 0.600).

### 3.7 Quality of life

The analysis of the QLQ‐C30 questionnaire scales indicated a tendency for higher general health status post‐intervention (80.6 vs. 70.8, p = 0.094). In the cognitive domain, a significant difference was observed between the baseline and follow‐up assessments (83.0 vs. 100.0, p = 0.020).

There were no significant differences in urinary symptoms between the assessments (p = 0.389). There were nonsignificant differences in the side effects of hormonal therapy (p = 0.114).

## 4 DISCUSSION

Our trial demonstrated the feasibility of recruiting and retaining participation among men with prostate cancer undergoing ADT and other comorbidities to a programme of supervised recreational football training in cooperation between clinical departments and sports clubs.

Exercise interventions counteracts ADT toxicity on physical functioning. Uth et al.,^11^ who investigated the effects of 32 weeks of recreational football, found that football improved physical functioning in tests requiring high power output (climbing performance, jump height and sit‐to‐stand test). In our study, the sit‐to‐stand test exhibited improvement from the baseline to the 4‐month evaluation (16.7 vs. 20.6 repetitions, p = 0.003), and further improvement was noted post‐intervention (16.7 vs. 23.7 repetitions, *p <* 0.001). This adaptation can likely be attributed to the frequent accelerations involved in football. Developing and sustaining muscle strength is crucial since reduced muscle strength is independently associated with impaired mobility and all‐cause mortality in older adults. Muscle strength, serving as an indicator of muscle quality, holds greater importance than muscle mass in assessing mortality risk.^18^ Agility also demonstrated significant differences between the baseline assessment, the 4‐month evaluation and post‐intervention. According to previous studies,^8^, ^19^ our programme improved balance from the baseline assessment to those performed at 4 and 8 months. These alterations are clinically important since ADT is associated with decreased functional performance, compromised dynamic balance and diminished bone density, resulting in an elevated susceptibility to falls and fractures.

Cardiorespiratory fitness is a strong independent predictor of cardiovascular morbidity and mortality in patients with CVD, cancer and the general population.^20^ Walking speed holds the potential as a predictor of future health status, functional deterioration and mortality.^21^ Different studies have independently demonstrated the predictive value of the 6MWT for both morbidity and mortality. Moreover, equations have been developed relating the 6MWT to peak oxygen consumption (peak VO_2_) for patients with cardiopulmonary diseases.^15^ We observed a significant improvement in aerobic capacity, with the patients improving the distances walked over 6 minutes (580.0 vs. 537.5, *p* = 0.005) with no significant difference observed in the measured rating of perceived exertion. There is a lack of studies investigating minimal clinically important differences (MCID) specifically in patients with prostate cancer undergoing ADT, thereby limiting our ability to contextualise our current results. However, a systematic review conducted by Bohannon et al. revealed that a change of MCID distance on 6MWT between 14.0 and 30.5 m may be clinically important across multiple patient groups.^22^

Previous research on men with prostate cancer has generally shown exercise to have no significant effect on blood pressure.^5^, ^8^, ^23^ A reduction of 10 mmHg in systolic blood pressure or 5 mmHg in diastolic blood pressure might decrease the risk of coronary heart events by 22% and stroke by 41%.^24^ In our study, two‐thirds of the patients were receiving medication for arterial hypertension. At 8 months of follow‐up, patients experienced a significant reduction in systolic blood pressure from a median of 134 to 123 and a non‐statistically significant decrease in diastolic blood pressure from a mean of 80 to 73 mmHg.

Previous studies have investigated the effect of exercise on metabolic biomarkers, such as blood lipids and blood glucose, in men on ADT for PCa but yielded inconsistent findings.^8^, ^23^ In our study, no differences were observed in the blood lipids, glucose control and high‐sensitive CRP.

The effects of exercise on body composition are complex and multifaceted. A recent systematic review and meta‐analysis of randomised trials indicate that exercise interventions for patients on ADT lead to increased lean body mass, reduced body fat mass and decreased body fat rate. The study revealed greater effectiveness for exercise lasting ≥ six months (vs. < six months), as well as for exercise initiated immediately after starting ADT (vs. delayed exercise).^25^ Surprisingly and in contrast with other studies,^8^, ^11^, ^12^, ^23^ no effects were observed on fat mass and lean body mass. First, it is important to highlight that in half of the patients, body composition assessment was not documented in the DXA scan performed after the intervention. Additionally, the volume of aerobic exercise performed might not have reached a level significant enough to cause notable changes in blood lipids, blood glucose and body fat considering the recommendation of five sessions per week for health maintenance and greater than 250 minutes per week for significant fat loss.^26^

A decline in BMD constitutes a significant adverse consequence of ADT, heightening the susceptibility to osteoporosis and fractures,^6^ and substantially compromising the patient's functional autonomy and quality of life. In agreement with a recent systematic review and meta‐analysis,^25^ our study failed to detect exercise‐related effects on BMD. The mechanical loading generated by resistance and impact training seems to act as a triggering factor in enhancing osteoblastic activity. In line with other authors,^10^, ^11^ we believe that the intermittent and impact nature of football characterised by variable impact forces acting on the bones from multiple angles, with intermittent accelerations and decelerations, plays a significant role in BMD. There was evidence suggesting that the diversity and dynamics of mechanical forces experienced during exercise are implicated in increasing bone mass.^27^ Uth et al.^11^ found that after 32 weeks of football training in men with PCa undergoing ADT there was an improvement of 0.8% and 1.0% in right and left total hip BMD, respectively, while in the control group, there was a decrease of −0.7% and −0.8%. Similarly, an improvement of 0.4% and 1.0%, in right and left femoral shaft BMD was observed, respectively, while the control group experience a decrease of −1.0 and −1.1%. In their study, Bjerre et al.^10^ revealed an improvement in hip BMD after 1 year, which was not evident at earlier time points, and notably, this change was found only in hip BMD did not extend to the spine. These findings are in line with the gradual process of bone remodelling and biochemical loading profile associated with football.^28^ However, the formation of new bone mass in this population presents challenges due to increased rates of age‐related endosteal bone resorption, coupled with androgen deprivation. Galvão et al.^8^ demonstrated that supervised aerobic and resistance exercise training of a moderate intensity improves EORTC QLQ‐C30 role and cognitive domain outcomes as well as symptom scales for fatigue, nausea and dyspnoea up to three months. Such interventions have also noted clinically relevant improvements in FACT‐P in men on long‐term ADT^5^ These results are supported by a systematic review that reported improvements in cancer‐specific QoL.^29^ Our study revealed a tendency for higher general health status post‐intervention (80.6 vs. 70.8, p = 0.094) and a significant difference in the cognitive domain (83.0 vs. 100.0, p = 0.020).

Currently, health services predominantly are still focused on the more conventional perspective regarding the treatment of patients with PCa, neglecting an integrated and holistic approach. Exercise is emerging as a promising supplementary treatment strategy in the oncology setting, with capacity to improve cardiorespiratory fitness, muscle strength, body composition and quality of life. Given the health benefits, the popularity and the feasibility of recreational football‐based exercise interventions of recreational football, this study's findings could indeed strengthen and widen this modality for the prevention, treatment and rehabilitation of PCa patients. Other strengths of our study include the assessment of clinically relevant outcomes with validated measures, and inclusion of patients with substantial disease burden.

There are several limitations that should be addressed. First, the small sample size of 12 patients and the absence of a control group precludes drawing firm conclusions. Additionally, patients were relatively well‐functioning individuals and showed strong motivation to engage in the programme, which might not accurately reflect all patients with PCa starting ADT.

## 5 CONCLUSIONS

Accumulating evidence suggests that exercise may ameliorate adverse effects related to ADT. This study contributes to current knowledge by demonstrating that a supervised recreational football‐based exercise programme can be implemented, improving physical fitness, aerobic capacity, systolic blood pressure and quality of life. Consequently, it could be considered as a viable alternative to conventional interventions in real life‐settings.

## AUTHOR CONTRIBUTIONS

*Conceptualization*: Miguel Silva‐Ramos and Sofia Mesquita. *Methodology*: Susana Póvoas, Sofia Mesquita, Diogo Nunes‐Carneiro, David Sá‐Couto, Mário Santos, and Miguel Silva‐Ramos. *Writing—original draft preparation*: Sofia Mesquita, Susana Póvoas, Miguel Silva‐Ramos, and Diogo Nunes‐Carneiro. *Writing—review and editing*: Susana Póvoas, Miguel Silva‐Ramos, Diogo Nunes‐Carneiro, and Avelino Fraga. *Supervision*: Miguel Silva‐Ramos and Susana Póvoas.

## ACKNOWLEDGEMENTS

We would like to thank all the participating patients, the Physical Education and Sports Sciences team, and the Departments of Urology, Cardiology, Oncology, and Nuclear Medicine. This work was supported by the Portuguese Association of Urology.

## CONFLICT OF INTEREST STATEMENT

The authors declare no conflict of interest.

## ETHICS APPROVAL AND CONSENT TO PARTICIPATE

The study was approved by the Ethics Committee of Education, Training and Research department at Unidade Local de Saúde de Santo António, Porto, Portugal. All participants signed an Informed Consent Statement.

## Supporting information


**Data S1.** Supporting Information
