# Supplementary material for: Impact of recreational football in men with prostate cancer undergoing androgen deprivation therapy
Source: BJUI Compass. 2025 Apr 10;6(4):e70008. doi: 10.1002/bco2.70008 (PMC11982628; doi:10.1002/bco2.70008)
Supplement: Supplementary file 1 — Data S1. Supporting Information [file BCO2-6-e70008-s001.docx]

**eMethods**

***Physical fitness***

*Flamingo balance test*

Postural balance was evaluated through a single-leg balance test. Patients were instructed to stand on one foot (dominant leg) with their eyes open on a 3cm wide and 5cm high metal bar for a duration of 1 minute. The timer was paused whenever patients lost their balance, prompting them to step down from the bar, and then resumed once balance was recovered. The count of falls before completing 1 minute in balance was recorded and used as a measure of postural balance.

*Agility*

Using a chair fixed to the floor, patients were instructed to sit in the middle of the chair with the back straight and a slightly inclined forward torso, placing their arms on thighs, and keeping their feet flat on the floor, one slightly ahead of the other. On a signal, patients stood up, walked as quickly as possible and then returned around a cone to a seated position. The timer was started at the “start” signal, whether the participant had or had not started the movement and stopped at the exact moment the patient sat down. The elapsed time between the “start” signal until the moment in which the participant sits on the chair was recorded.

*Sit-to-stand test*

Using a chair fixed to the floor with a seat 45cm above the floor, patients were instructed to sit in the middle of the chair with the back straight, arms crossed over their chest, and feet flat on the floor. On a signal “start”, patients stood up fully and then returned to a seated position, as many times as possible in 30 seconds. Correct technique was demonstrated prior to the test. Patients practised two or three repetitions before the start of the test.

*Aerobic capacity*

Functional capacity was estimated using the 6-minute walk test (6MWT). Patients were instructed to walk for 6 minutes along a 50-meter flat, straight, and hard-surfaced corridor. The walking course was marked every 5 meters and cones were placed in the turnarounds. During the test, participants were encouraged to walk at a pace suitable for their condition and they were allowed to stop or slow down if needed, resuming walking as soon as possible. The number of laps and the additional distance covered were recorded and the 6MWD was calculated. Patients were instructed to classify their rating of perceived exertion (namely global, muscular and respiratory) during the test, according to a 0-10 AU scale. Heart rate was monitored during the test. The maximal HR and the HR values at 30, 60 and 120 seconds after the end of the test were recorded. This test can be used as a predictor of functional and objective (VO_2_max) fitness (15).

*Physical activity and sedentary time*

Daily physical activity and sedentary time were measured using the International Physical Activity Questionnaire (16).

***Quality of life***

The tools with the best evidence for psychometric properties and feasibility for use in routine practice and research settings to assess patient-reported outcome measures in patients with PCa were European Organisation for Research and Treatment of Cancer (EORTC) QLQ-C30 (17) and QLQ-PR25 (18) .

The EORCT QLQ-C30 version 3.0 questionnaire is a general module for examining the quality of life of cancer patients without taking account the type, stage and location of neoplasm. The questionnaire consists of 30 questions which are divided into 3 main parts – global health status/QoL scale; five functional scales (physical, role, cognitive, emotional, and social) and symptom scales (fatigue, pain, nausea and vomiting, additional symptoms commonly reported by cancer patients such as dyspnoea, loss of appetite, insomnia, constipation and diarrhoea and perceived financial impact of the disease). The questions are answered on a 4-point scale (1 – not at all, 2 – little, 3 – much, 4 – very much) assessing the intensity of the analysed parameter. The only exceptions are the last two questions of the questionnaire, which concern a general assessment of health, where a 7-point scale is used.

The EORCT QLQ-PR25 questionnaire is a supplementary to the EORCT QLQ-C30 questionnaire and incorporates five multi-item scales to assess sexual activity, sexual functioning, urinary symptoms, bowel symptoms, and hormonal treatment-related symptoms.

All the scales and single-item measures range in score from 0 to 100. In the EORCT QLQ-C30 questionnaire, obtaining a higher score for the functional scales and the general health scale indicates a higher quality of life while the higher the score on the symptomatic scales, the greater severity of symptoms. For the EORCT QLQ-PR25 questionnaire, a higher score on the functional scales means a higher level of functioning, but on the symptomatic scales, a higher score means a greater severity of symptoms.
